# Accelerating String Set Matching in FPGA Hardware for Bioinformatics Research

**DOI:** 10.1186/1471-2105-9-197

**Published:** 2008-04-15

**Authors:** Yoginder S Dandass, Shane C Burgess, Mark Lawrence, Susan M Bridges

**Affiliations:** 1 Institute of Digital Biology, Mississippi State University, Mississippi, 39762, USA; 2 Department of Computer Science and Engineering, Mississippi State University, Mississippi, 39762, USA; 3 Life Science and Biotechnology Institute, College of Veterinary Medicine, Mississippi State University, Mississippi, 39762, USA; 4 Department of Basics Science, Mississippi State University, Mississippi, 39762, USA

## Abstract

**Background:**

This paper describes techniques for accelerating the performance of the string set matching problem with particular emphasis on applications in computational proteomics. The process of matching peptide sequences against a genome translated in six reading frames is part of a proteogenomic mapping pipeline that is used as a case-study. The Aho-Corasick algorithm is adapted for execution in field programmable gate array (FPGA) devices in a manner that optimizes space and performance. In this approach, the traditional Aho-Corasick finite state machine (FSM) is split into smaller FSMs, operating in parallel, each of which matches up to 20 peptides in the input translated genome. Each of the smaller FSMs is further divided into five simpler FSMs such that each simple FSM operates on a single bit position in the input (five bits are sufficient for representing all amino acids and special symbols in protein sequences).

**Results:**

This bit-split organization of the Aho-Corasick implementation enables efficient utilization of the limited random access memory (RAM) resources available in typical FPGAs. The use of on-chip RAM as opposed to FPGA logic resources for FSM implementation also enables rapid reconfiguration of the FPGA without the place and routing delays associated with complex digital designs.

**Conclusion:**

Experimental results show storage efficiencies of over 80% for several data sets. Furthermore, the FPGA implementation executing at 100 MHz is nearly 20 times faster than an implementation of the traditional Aho-Corasick algorithm executing on a 2.67 GHz workstation.

## Background

*String set matching *is an important operation in computational biology. For example, when proteomics data is used for genome annotation in a process called proteogenomic mapping [1-5], a set of peptide identifications obtained using mass spectrometry is matched against a target genome translated in all six reading frames. Given a large number of peptides and long translated genome strings, the fundamental problem here is to efficiently search for a large set of *pattern *strings (*i.e*., the set of peptides) in a larger *text *string (*i.e*., the translated genome).

Efficient substring search algorithms such as Boyer-Moore [6] and Knuth-Morris-Pratt [7] that locate single pattern strings within a larger text string can be used in a multi-pass manner (*i.e*., one pass for each string in the set of peptides). However, this approach does not scale well with an increasing number of pattern strings. In particular, assuming *p *patterns with an average length of *n *and a text string of length *m*, naïve, multi-pass, approaches have computational complexity of *O*(*p*(*m *+ *n*)).

The Aho-Corasick algorithm [8] provides a scalable solution to the string set matching problem in that it incorporates the search mechanism for the entire set of patterns into a single finite state machine (FSM). The power of Aho-Corasick stems from the ability of the algorithm to find the location of the strings in the pattern set in the text string in a single pass. The computational complexity of Aho-Corasick search is *O*(*m *+ *k*) where *k *is the total number of occurrences of the pattern strings in the text. This linear processing time complexity has resulted in the widespread use of Aho-Corasick in string matching applications.

The performance of the Aho-Corasick algorithm can be further enhanced by implementing it in hardware. Tan and Sherwood [9]were the first to describe an area-efficient hardware approach for implementing the Aho-Corasick for network intrusion detection systems implemented in application specific integrated circuits (ASICs). However, the complexity and costs associated with ASIC development is a significant impediment in their adoption in computational biology. Field programmable gate array (FPGA) devices, on the other hand, can be repeatedly reconfigured to create a variety of application-specific processing elements. This reconfigurable nature makes FPGAs a popular low-cost alternative to the development of specialized ASICs for a variety of application domains, including computational biology.

Although the fundamental Aho-Corasick algorithm is identical for all string set matching applications, optimization for specific applications and target hardware results in significant performance and storage efficiencies. The main contribution of this paper is a case-study demonstrating how an Aho-Corasick architecture and finite state machine (FSM) organization can be specifically optimized for incorporation into proteogenomic pipeline using field programmable gate array (FPGA) hardware. This paper also demonstrates how the 18-kbit random access memory (RAM) blocks available on Xilinx's Virtex-4 FPGAs and 9-kbit RAM blocks available in Altera's FPGAs can be utilized to create resource-efficient amino acid sequence set matching engines. Furthermore, the use of RAM instead of FPGA logic resources for encoding FSM state transitions enable the FPGA to be quickly reconfigured for matching different peptide sets.

### Related Work

The Aho-Corasick algorithm (ACA) is widely used in computational biology for a variety of pattern matching tasks. For example, Brundo and Morgenstern use a simplified version of ACA to identify anchor points in their CHAOS algorithm for fast alignment of large genomic sequences [10,11]. The TROLL algorithm of Castelo, Martins, and Gao uses ACA to locate occurrences of tandem repeats in genomic sequence [12]. Farre et al. use Aho-Corasick as the search algorithm for predicting transcription binding sites in their tool PROMO v. 3. [13] Hyyro et al. demonstrate that Aho-Corasick outperforms other algorithms for locating unique oligonucleotides in the yeast genome[14]. The SITEBLAST algorithm [15] employs the Aho-Corasick algorithm to retrieve all motif anchors for a local alignment procedure for genomic sequences that makes use of prior knowledge. Sun and Buhler use Aho-Corasick deterministic finite automata (DFA) to design simultaneous seeds for DNA similarity search [16]. The AhoPro software package adapts the Aho-Corasick algorithm to compute the probability of simultaneous motif occurrences [17].

There has been a good deal of attention in the use of FPGAs to address bottlenecks in computational biology pipelines. Examples include the use of FPGAs to improve the speed of homology search [18,19] for computing phylogenetic trees [20], for the pairwise alignment step in multiple sequence alignment using CLUSTALW [21], and for acceleration of the Smith-Waterman sequence alignment algorithm [18]. In computational proteomics, Alex, et al. [22] have demonstrated the use of FPGAs to accelerate peptide mass fingerprinting. (Note that one step in their algorithm is similar to ours except they translate the peptide in all possible ways in all six reading frames and compare to a nucleotide sequence.) Bogdan et al. [23] have applied FPGAs to the problem of analyzing mass spectrometric data generated by MALDI-ToF instruments by developing hardware implementations of algorithms for de-noising, baseline correction, peak identification, and deisotoping.

Hardware implementations of ACA have been developed for applications other than bioinformatics. Snort is a popular computer security program that looks for a set of "signature" patterns corresponding to known intrusion attacks in network packets. Tan and Sherwood [9] split the Aho-Corasick implementation for Snort into four separate FSMs such that each FSM is responsible for two separate bit positions in the signature string set and network packet. This *bit-split *implementation is more efficient in terms of hardware area. However, their paper does not exploit the availability of specialized hardware resources in FPGAs.

Jung, Baker and Prasanna [24] describe an implementation of the bit-split Aho-Corasick algorithm for Snort using field programmable gate array (FPGA) technology. They optimize the bit-split implementation of Aho-Corasick for Snort by using RAM blocks available on Xilinx FPGAs. However, in Snort, the input alphabet consists of all 256 distinct symbols that can be represented using 8 bits in a byte. In string-matching for proteogenomic mapping, the alphabet consists of 20 amino acids and a small number of additional symbols that can be represented in five bits. Furthermore, Jung *et al*. do not exploit the dual-ported nature of RAM blocks in modern FPGAs that enables more efficient utilization of storage resources. Therefore, the previously described bit-split implementations designed for Snort are not optimal for proteomics processing in FPGAs.

Sidhu and Prasanna describe a technique for constructing non-deterministic finite state automata (NFA) from regular expressions that can be used for string matching [25]. Their solution requires *O*(*n*^2^) space where *n *is the number of characters in the regular expressions to be searched. Because their NFA is implemented entirely in FPGA logic, this technique requires large FPGAs in order to implement searches for large string sets.

Lin *et al*. describe a technique for improving the space efficiency by up to 20% for NFA implementations in FPGA logic fabric [26]. Their architecture optimizes space by sharing common prefixes, infixes, and suffixes between multiple regular expressions.

Fide and Jenks provide an extensive survey of string matching techniques and implementations in hardware [27]. The survey focuses on intrusion detection and network router implementation.

## Methods

### The Aho-Corasick Algorithm

The Aho-Corasick algorithm consists of an initial preprocessing phase that creates the FSM from the set of pattern strings. The FSM resulting from the preprocessing phase is subsequently used for performing the string set matching. The preprocessing phase has a runtime complexity of *O*(*pn*) and the search phase has a runtime complexity of *O*(*m *+ *k*). Detailed description and analysis of Aho-Corasick can be found in [8]. A brief description follows below.

In the preprocessing phase, the FSM is constructed using two steps. In the first step, a set of target strings is organized into a "keyword" tree. The root of the tree represents the state when no part of any pattern string has been found in the input message. The remaining nodes of the tree represent states where the pattern strings have been partially or fully matched. The edges in the tree represent the transitions resulting from the occurrence of specific symbols in the text string. The path from the root node to any node on the tree represents the subset of pattern strings that are potential matches.

In the second preprocessing step, "failure links" are added to the tree. Failure links lead from nodes on one branch of the tree to nodes on other branches. Failure links are needed because patterns strings can overlap in the text string and when the current branch of the tree fails to produce a match because of the current symbol in the text string, the FSM needs to resume processing from a new branch, without having to rescan input symbols.

**Figure 1:**
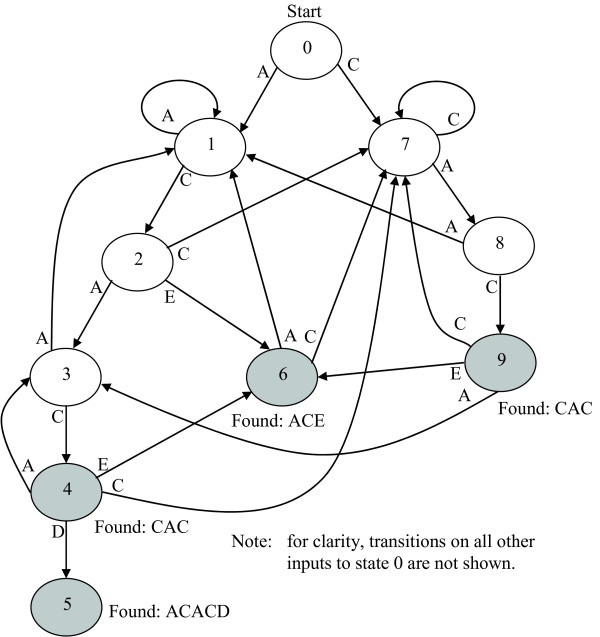
An FSM for matching peptide set {ACACD, ACE, CAC}.

In a computer, the FSM state transitions can be represented in the form of a table. Figure 1 illustrates the organization of the FSM for an implementation of Aho-Corasick that matches the peptide set {ACACD, ACE, CAC}; Table 1 presents a table-oriented representation used for implementation of the same FSM. In Figure 1, the state 0 is the start state and the shaded states 4, 5, 6, and 9 match peptides CAC, ACACD, ACE, and CAC, respectively. When the FSM is in any state and receives an input symbol not shown in the figure, the FSM transitions to state 0. At runtime, the FSM interpreter reads the row corresponding to the current state from the table, reads the next input symbol from the reading frame, and determines the next state from the row entry corresponding to the input symbol. When the FSM transitions to as state, it looks at the *pattern match *column of the table's corresponding row in order to determine if a match has occurred. A non-null entry in the *pattern match *entry of row specifies the pattern that has been located by the FSM.

**Table 1:** A table-oriented representation of the FSM for peptide set {ACACD, ACE, CAC}.

	**Input Text Symbol**								
**Current State**	**A**	**B**	**C**	**D**	**E**	**F**	**...**	**Z**	**Match**

**0**	1	0	7	0	0	0	0,0,...,0	0	Ø
**1**	1	0	2	0	0	0	0,0,...,0	0	Ø
**2**	3	0	7	0	6	0	0,0,...,0	0	Ø
**3**	1	0	4	0	0	0	0,0,...,0	0	Ø
**4**	3	0	7	5	6	0	0,0,...,0	0	Pep2
**5**	1	0	7	0	0	0	0,0,...,0	0	Pep1
**6**	1	0	7	0	0	0	0,0,...,0	0	Pep3
**7**	8	0	7	0	0	0	0,0,...,0	0	Ø
**8**	1	0	9	0	0	0	0,0,...,0	0	Ø
**9**	3	0	7	0	6	0	0,0,...,0	0	Pep2

### The Bit-Split Aho-Corasick Implementation

The branching factor of the Aho-Corasick FSM tree depends on the number of symbols possible in the input text string. For example, the branching factor is 256 when eight bits are used for representing the alphabet of valid symbols in the strings (as is the case with intrusion detection applications such as Snort). However, because only five bits are needed for representing all the 20 amino acids and additional special symbols (*e.g*., those representing ambiguous amino acids), the size of the table can be reduced significantly by reducing the total number of columns.

Additional savings in storage can be obtained by splitting the FSM into smaller FSMs. Following are two approaches to make an FSM smaller:

1. Reduce the number of peptides in the peptide set. This will reduce the number of states in the FSM, and therefore, will reduce the number of bits required to store the "next state" transition value.

2. Split the FSM into simpler FSMs that are responsible for encoding and operating on individual bit positions of the symbols in the peptides patterns and the text string. For example, the FSM in Table 1 can be split into five separate bit-split FSMs, *FSM*_0_, *FSM*_1_, *FSM*_2_, *FSM*_3_, *FSM*_4_, one for each of the five bit positions it takes to encode all the peptides. Because the bit-split FSMs operate independently from each other, all of the separate bit-split FSMs must agree on a match before a peptide match is confirmed.

The general bit-split FSM algorithm is described in detail in [24]. The bit split FSM process is described below for the FSM in Table 1, resulting in the five bit-split FSMs designated as *FSM*_0_, *FSM*_1_, *FSM*_2_, *FSM*_3_, and *FSM*_4 _shown in Tables 3, 4, 5, 6, and 7, respectively. Consider the construction of *FSM*_0_, the bit-split FSM corresponding to bit position 0; table 2 describes the bitwise encoding of selected amino acids. State *n *in the original FSM is designated as FSM:*n *and state *m *in *FSM*_0 _is designated as *FSM*_0_:*m*.

**Table 2:** Bit encoding of selected peptides

	**Bit encoding of selected peptides**				
**Peptide**	**4**	**3**	**2**	**1**	**0**

A	0	0	0	0	0
C	0	0	0	1	0
D	0	0	0	1	1
E	0	0	1	0	0
...	...	...	...	...	...
M	0	1	1	0	1
...	...	...	...	...	...
Y	1	1	0	0	1

**Table 3:** The Bit-Split FSM corresponding to bit position 0 (*FSM*_0_)

**State**	**0**	**1**	**Match**
**0**	1	0	Ø:000
**1**	2	0	Ø:000
**2**	3	0	Ø:000
**3**	4	0	3,2:110
**4**	4	5	3,2:110
**5**	1	0	1:001

**Table 4:** The Bit-Split FSM corresponding to bit position 1 (*FSM*_1_)

**State**	**0**	**1**	**Match**
**0**	1	2	Ø:000
**1**	1	3	Ø:000
**2**	4	2	Ø:000
**3**	5	2	Ø:000
**4**	1	6	Ø:000
**5**	1	7	2:010
**6**	5	2	3:100
**7**	5	8	3:100
**8**	4	2	1:001

**Table 5:** The Bit-Split FSM corresponding to bit position 2 (*FSM*_2_)

**State**	**0**	**1**	**Match**
**0**	1	0	Ø:000
**1**	2	0	Ø:000
**2**	3	4	Ø:000
**3**	5	4	3:100
**4**	1	0	2:010
**5**	6	4	3:100
**6**	6	4	3,1:101

**Table 6:** The Bit-Split FSM corresponding to bit position 3 (*FSM*_3_)

**State**	**0**	**1**	**Match**
**0**	1	0	Ø:0
**1**	2	0	Ø:0
**2**	3	0	Ø:0
**3**	4	0	3,2:110
**4**	5	0	3,2:110
**5**	5	0	3,2,1:111

**Table 7:** The Bit-Split FSM corresponding to bit position 4 (*FSM*_4_)

**State**	**0**	**1**	**Match**
**0**	1	0	Ø:0
**1**	2	0	Ø:0
**2**	3	0	Ø:0
**3**	4	0	3,2:110
**4**	5	0	3,2:110
**5**	5	0	3,2,1:111

Initially, the root node *FSM*_0_:0 is added to *FSM*_0_. Next, all states in the original FSM that can be reached from *FSM*:0 (the root node from the original FSM) when the bit position in the transition is 0 are determined and aggregated into a new bit-split node *FSM*_0_:1. In the example, *FSM*:1 and *FSM*:7 are aggregated to form *FSM*_0_:1. Because *FSM*_0_:1 does not already exist in *FSM*_0 _(*i.e*., there in no state in *FSM*_0 _that is aggregated from *FSM*:1 and *FSM*:7), it is added to *FSM*_0 _with a transition from *FSM*_0_:0 when the input bit is 0. Next, all states in the original FSM that can be reached from *FSM*:0 when the bit position in the transition is 1 are determined and aggregated; in this example, there are no such states. Therefore, the transition from *FSM*_0_:0 goes back to *FSM*_0_:0 when the input bit is 1. This process is repeated for all newly added states in *FSM*_0_.

*FSM*_0_:1 was added previously and is examined next. Note that *FSM*_0_:1 is an aggregate of *FSM*:1 and *FSM*:7. Therefore, all states in the original FSM that can be reached from either *FSM*:1 or *FSM*:7 when the input at bit position is 0 are aggregated into *FSM*_0_:2. In this example, *FSM*_0_:2 is created from *FSM*:2 and *FSM*:8. Because *FSM*_0_:2 does not already exist in *FSM*_0_, it is added to *FSM*_0 _with a transient of 0 from *FSM*_0_:1. Again, there is no transition from *FSM*_0_:1 when the input bit is 1, therefore, state *FSM*_0_:1 transitions back to *FSM*_0_:0 when the input bit is 1.

This process is continued until there are no new states added to *FSM*_0_. Note that only unique new nodes are added to *FSM*_0_. When a new node *FSM*_0_:*n *is created by aggregation but another node, *FSM*_0_:*k*, created by aggregating the same set of nodes already exists in *FSM*_0_, then instead of inserting the new node, *FSM*_0_:*n*, a transition to *FSM*_0_:*k *is inserted into *FSM*_0_. Peptide matches are also handled using aggregation (*i.e*., state *FSM*_0_:*k *matches all the peptides that are matched by the states in the original FSM that were aggregated into *FSM*_0_:*k*). This process is repeated for all bit positions resulting in the five separate bit-split FSMs depicted in tabular form in Tables 3, 4, 5, 6, and 7.

Because several states from the original FSM that match different peptides may be combined into a single state in a bit-split FSM, a mechanism to indicate multiple matches is required. In the bit-split FSM, a vector of bits is used to encode the peptide matching attribute of for each state. For example, state *FSM*_0_:3, matches peptides 2 and 4, and therefore, has a peptide matching bit vector containing 110. Using this mechanism, after the various state machines enter their respective new states, a bitwise logical *and *operation can be used to determine the peptide match that all five FSMs agree on. For example, assume that a some point in time, the bit-split FSMs in Tables 3, 4, 5, 6, and 7 are in states *FSM*_0_:3, *FSM*_1_:7, *FSM*_2_:6, *FSM*_3_:5, and *FSM*_4_:5 with match bit vectors of 110, 100, 101, 111, and 111, respectively. The bitwise logical *AND *of the five match bit vectors results in the bit vector 100 that indicates that peptide 3 is matched in this case. However, if *FSM*_0 _is in state *FSM*_0_:5, with a matching bit vector of 001, then the result of the logical *and *will result in 000, indicating that no peptides are currently matched.

### FPGA Implementation

The Aho-Corasick algorithm can be implemented in an FPGA by directly using the table representation depicted in Tables 3, 4, 5, 6, and 7. Implementing the bit-split state machines in this manner (*i.e*., using lookup tables) is more resource efficient as compared with encoding sequences of conditional state transitions in the FPGA logic fabric. In modern FPGAs, the tables can be stored using either configurable logic block resources (*i.e*., distributed RAM) or blocks of random access memory (*i.e*., BRAM). However, BRAM is more efficient when storing large tables because it has higher storage density than distributed RAM [9].

Xilinx FPGAs provide a large number of 18-kbit BRAMs that can be organized into 512 rows of 36-bit wide words [28]. The Xilinx BRAMs are dual ported; therefore, by tying the high order bit of the 9-bit BRAM address input to 0 on one port and to 1 on the other port, the BRAM can be divided into two independent 9-kbit RAM blocks containing 256 rows of 36-bit words each. Altera FPGAs also provide a large number of 9-kbit BRAMs that can be organized into 256 rows of 36-bit wide words (other BRAM configurations are also possible but are not useful in this application) [29].

A 256 by 36 bit block of RAM can hold 256 rows of a bit-split Aho-Corasick FSM. Recall that the bit-split FSM reads the row corresponding to the current state in order to output the peptide match bit vector and to determine the next state. The FSM can transition into one of two states (note that the FSM can transition back into the state it is currently in) depending on the input value (*i.e*., 0 or 1). Because 8 bits are required to represent each of the 256 possible *next state *values, 16 bits in each 36-bit wide row are used for storing the two possible next state values. The remaining 20 bits in the row are used to store the 20 position peptide match bit vector.

**Figure 2:**
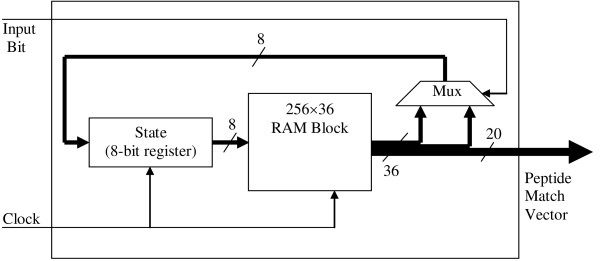
Bit-Split Aho-Corasick FSM Architecture.

**Figure 3:**
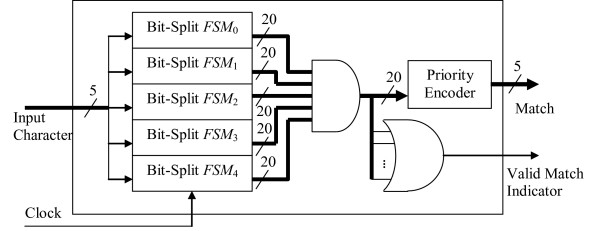
Aho-Corasick Tile Architecture.

Figure 2 illustrates the architecture of a bit-split FSM. In addition to the 9-kbit RAM block, the implementation requires an 8-bit register to store the current state and a multiplexer to select one of the two next state values based on the value of the input bit. Five of these bit-split FSM modules are combined in order to create a complete Aho-Corasick *tile *as depicted in Figure 3. Inside a tile, the 5-bit input to the Aho-Corasick implementation is distributed to the five bit-split FSMs. A bit-wise and operator combines the bit-split peptide match vectors into the consensus 20-bit peptide match vector output.

In order to conserve signal routing resources, the consensus peptide match vector is converted into a 5-bit numerical value using a 20-to-5 bit priority encoder (the reason for using a priority encoder is provided towards the end of this section). The encoder essentially scans the consensus peptide match vector in increasing index order and returns the index of the first bit that has a value of 1. Therefore, peptides that appear near the beginning of the list of peptides have higher priority than those appearing later. If all consensus peptide match vector bits are clear (*i.e*., there is no match), the priority encoder returns an undefined value. Therefore, in order to indicate that a peptide has been found, a *valid *output signal is also generated when any of the consensus peptide match vector bits are set.

**Figure 4:**
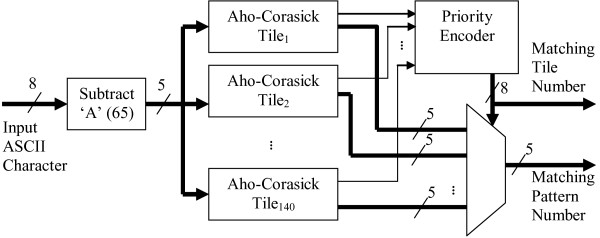
Aho-Corasick Implementation Architecture using 140 tiles (clock and reset signals are not shown for clarity).

Typically, proteomics pipelines require the detection of more than 20 peptides. In this case, several Aho-Corasick tiles can be utilized in parallel as shown in Figure 4. The input reading frame is simultaneously streamed to all tiles. The output of the tiles is combined into a single output peptide number using a priority encoder. Because the priority encoder produces an undefined value when no peptide is matched, a *match *indicator signal is also generated when any of the tiles indicate a valid match.

Using the architecture described above, each tile can detect up to 20 peptides in an input stream of reading frame data. However, because the tile has a capacity of only 256 states per bit-split FSM, in some cases, it may be necessary to reduce the number of peptides that can be detected in order to create a bit-split FSM with no more than 256 states (note that all five peer bit-split FSMs must represent the same reduced number of peptides). A simple iterative greedy algorithm can be employed to allocate peptides to tiles using a trial-and-error approach. Initially, the algorithm assigns a set of 20 peptides to a tile. If the bit-split FSM for the given number of peptides has more than 256 states, the algorithm reduces the number of peptides assigned to the tile and tries again until the bit-split FSM is successfully created.

In order to minimize the number of states required in the Aho-Corasick implementation, strings beginning with the same sequence of characters should be grouped together into the same tile. This is because the strings will share the same initial states in the FSM. One way to achieve this is to sort the set of peptide strings in ascending alphabetical order before being assigned to the Aho-Corasick tiles.

Additionally, this bit-split Aho-Corasick implementation architecture can only indicate a single peptide match at any given time. This is typically not a problem unless one peptide is a *suffix *of another peptide. Peptide *p*_*s *_is a suffix of peptide *p *if and only if the length of *p *is greater than or equal to the length of *p*_*s *_and *p *ends with a substring that is identical to *p*_*s*_. In this case, if the string for *p *appears in the text, it is sufficient to simply indicate that *p *has been found because this also implies that *p*_*s *_has been found. The priority encoding architecture ensures that the detection of a match with *p *receives higher priority than *p*_*s *_as long as *p *appears before *p*_*s *_in the sorted set of peptide strings. Therefore the sorting of the peptide string set must account for both alphabetical and suffix-based priority ordering.

### Analysis of FSM Storage Utilization Efficiency

Assume that *P*_*i*_, 1 = *P*_*i *_= 20, is the number of peptides that can be detected by tile *i*. This means that in each bit-split FSM table row, 20 – *P*_*i *_bits are unused for indicating matches. Furthermore, in a majority of cases, a bit-split FSM requires fewer than 256 states to detect *P*_*i *_peptides. This means that when FSM_*b *_of tile *i *requires *S*_*i*,*b *_states, such that 1 <*S*_*i*,*b *_= 256, then 256 – *S*_*i*,*b *_rows of available storage in the 9-kbit RAM block are unused.

The storage utilization efficiency of a single 256 by 36 bit block used by a single bit-split FSM is computed as follows:

(1) $$\eta_{i,b}=\frac{16S_{i,b}+S_{i,b}P_{i}}{256\times 36}=\frac{S_{i,b}\left(16+P_{i}\right)}{9216}.$$

The overall storage utilization for an implementation requiring *T *tiles can be computed using the following expression:

(2) $$\eta =\frac{\sum_{i=1}^{T}\sum_{b=0}^{4}S_{i,b}(16+P_{i})}{5\times 9216\times T}=\frac{\sum_{i=1}^{T}\sum_{b=0}^{4}S_{i,b}(16+P_{i})}{\text{46080}T}.$$

## Results and Discussion

The bit-split Aho-Corasick algorithm was implemented on the Xilinx Virtex-4 FX-100 FPGA. This device has 376 18-kbit BRAM blocks, of which 350 are used for implementing Aho-Corasick tiles and the remaining 26 are reserved for meeting the storage requirements of other modules that support the implementation (*e.g*., I/O functions and the memory for the embedded processor core that controls the overall implementation).

Recall that a Xilinx BRAM can be configured as two 9-kbit RAM blocks. This means that there are effectively 700 RAM blocks available that can hold a total of 140 tiles. Because each tile requires five 9-kbit RAM blocks and can search for at most 20 peptides, the maximum number of peptides that can be searched using this device is 700 × 20/5 = 2,800. Note that larger FPGA devices can be used to search for more than 2800 peptides in a single pass. For example, the Virtex-4 FX-140 has 552 BRAM blocks that can be configured to hold over 4,000 peptides. For the experiments, sets of 2,800 peptides were derived by performing in-silico trypsin digestion on reading frames corresponding to chromosome 1 of the human genome. The maximum size of the peptides was fixed at 30 amino acids. However, in order to measure the effect of peptide size on the storage efficiency, the minimum size was varied in order to produce 100 unique sets each of peptides with minimum sizes of 5, 10, 15, and 20.

Table 8 summarizes the results from generating the Aho-Corasick implementation for the various peptide sets. Most of the peptide sets where the minimum peptide size is 5 and having an average length of just over 11 amino acids were accommodated using 140 tiles. Two of these peptide sets require an additional tile because for each of these sets one of the tiles can only accommodate 19 peptides within the 256 state limit. The average storage utilization in the tiles is approximately 52.7% because many of the bit-split FSMs require significantly fewer than the available 256 states.

**Table 8:** Tile packing efficiency result

**Peptide Length**			**Average Number of Tiles Required**	**Average Peptides Per Tile**	**Average Storage Efficiency**
**Min**	**Max**	**Average**			
5	30	11.28	140.02	19.99	52.70%
10	30	15.40	141.41	19.80	81.12%
15	30	19.79	178.40	15.70	81.53%
20	30	23.70	277.23	12.32	72.96%

The number of tiles required for the peptide sets with the minimum peptide length of 10 amino acids (average length of 15.40) varies between 141 and 142 with an average of 19.8 peptides detected per tile. The average storage utilization is a much higher 81.12%.

The average number of tiles required for the peptide sets with the minimum peptide length of 15 amino acids (average length of 19.79) is 178.40 with an average of 15.7 peptides detected per tile. The average storage utilization is relatively high at 81.53%. This efficiency is comparable to the efficiency of the peptide sets with average size of 15.40. However, while in the case of the shorter peptides, underutilization of row storage is the main cause of inefficiency, for longer peptides, underutilization of the peptide match vector storage has a larger contribution to the overall inefficiency.

The average number of tiles required for the peptide sets with the minimum peptide length of 20 amino acids (average length of 23.70) is 277.23 with an average of 12.32 peptides detected per tile. The number of tiles required is significantly larger than in the previous cases because the bit-split FSMs have more states. The storage efficiency is also reduced to 72.96% because of significant underutilization of match vector storage.

The runtime performance of the FPGA-based bit-split Aho-Corasick implementation was compared to the performance of the Aho-Corasick implementation on a standard workstation. The bit-split Aho-Corasick design with supporting elements such as an embedded PowerPC processor, an ATA hard disk controller, an RS232 link, system busses, and memory are synthesized to run at a clock frequency of 100 MHz. The ATA disk controller is used for reading data at a peak rate of 100 MB/s (*i.e*., one character from the reading frame is streamed to the Aho-Corasick tiles every clock cycle). The PowerPC is responsible for initializing the disk drive and initiating the read operations. The PowerPC also monitors the peptide match indications from the tiles and reports match data (e.g., peptide and location) to the host workstation over the RS232 link.

Simulation results show that the Aho-Corasick tiles can operate at frequencies over 150 MHz, resulting in input rates exceeding 1.2 gigabits per second. Although the Aho-Corasick tiles can operate at faster frequencies, in this series of experiments the clock frequency was restricted to execute at 100 MHz system clock in order to eliminate the complexity that arises with designs containing multiple clock domains. Essentially, the Aho-Corasick tiles operate at 100 MHz in order to match the ATA controller's peak data delivery rate of 100 MB/s. Note that the tiles do not introduce any processing delays (*i.e*., the disk drive is the primary performance bottleneck in this implementation and increasing the implementation's clock frequency to 150 MHz produces no tangible improvements in processing time). Furthermore, in order to minimize processing and concomitant delays associated with a file system, the reading frame data is stored on consecutive sectors on a raw disk (*i.e*., the disk is not formatted using a well-known, operating system supplied, file system).

Reading frame data is derived using software on a standard workstation by concatenating all the chromosomes in the human genome (separated by sequences of 100 'N' characters) and subsequently translating the concatenated genome data into reading frames. The six resulting reading frames are also concatenated together, giving 6,160,844,220 bytes of text to be searched for a set of 2,800 peptides. The reading frame data is written to 12,032,899 consecutive sectors on an IDE disk drive, at a known starting location. A database of reading frame data for various genomes can be maintained in a similar manner on a large disk drive.

For testing the performance of the FPGA-based implementation, the disk drive containing the reading frame data is connected to the FPGA board. A flash RAM module containing the Aho-Corasick implementation configuration file and RAM block content implementing the FSM is also connected to the board. On bootup, the FPGA board reads the configuration information from the flash RAM and begins executing the Aho-Corasick algorithm.

For these experiments, a set of 2,800 peptides that fit in exactly 140 tiles (*i.e*., a set with minimum and average peptide lengths of 5 and 11.5257 amino acids, respectively) was selected. Note that storage efficiency of the selected peptide set has no bearing on the runtime performance of the bit-split Aho-Corasick implementation. This is because the Aho-Corasick tiles each search for a subset of 20 peptides in parallel. The performance of the FPGA-based implementation was compared with the performance of a software implementation employing a traditional table-driven Aho-Corasick organization in which a single large table represents a single FSM with all the states for all 2,800 peptides. The software implementation was executed on a Windows XP workstation having a 2.67 GHz Intel Core2 Duo processor, 2 GB RAM, and a pair of Serial ATA disks configured as a RAID 0 disk drive (*i.e*., striped data for fast disk I/O), formatted as an NTFS volume.

Five runs each of the FPGA and workstation implementations were performed. The FPGA implementation takes, on average, 94.17 seconds to process the entire 6 gigabytes of reading frame data. The workstation implementation takes an average of 1870.18 seconds to complete the search. This means that the FPGA-based implementation is nearly 20 times faster than the workstation implementation.

Implementations of larger designs in FPGAs typically have lower operating frequencies as compared with smaller designs. Therefore, in order to study the practical limits of implementations with large numbers of tiles, Xilinx's FPGA application development tool, XST 9.1, was used to implement a number of designs with varying number of Aho-Corasick tiles for a hypothetical board containing the Virtex-4 FX-140 device. Table 9 lists the performance statistics of the designs reported by XST. The smallest design composed of 40 tiles, requiring 100 BRAM blocks with a capacity of 800 peptides operates at a frequency of 177.054 MHz. The largest design composed of 200 tiles, requiring 500 BRAM blocks with a capacity of 4,000 peptides operates at a frequency of 134.971 MHz. Clearly, even the largest design meets the 100 MHz frequency requirement.

**Table 9:** Operating frequencies of Aho-Corasick designs with a variety of tiles on Virtex-4 FX-140

**Peptides**	**Tiles**	**BRAMs**	**Frequency (MHz)**
800	40	100	177.054
1,600	80	200	166.030
2,400	120	300	167.954
3,200	160	400	132.503
4,000	200	500	134.971

## Conclusion

This paper describes a technique for accelerating string set matching implementation using FPGAs for use in proteomics processing pipelines. FPGAs provide a large number of embedded memory blocks that enable more efficient implementation of FSMs than possible using the FPGA logic fabric. Furthermore, the synthesized tile-based design can be reused for different peptide sets by simply initializing the RAM block content with appropriate bit-split FSM state data. This is much faster than having to rerun the significantly time consuming "placing and routing" synthesis stages required for logic-based implementations in FPGA fabric for each new peptide set.

Empirical results show that the FPGA-based implementation outperforms the workstation implementation by a factor of 20. This result shows that using specialized hardware to solve the string set matching problem can make a significant impact on the runtime of a number of computational biology processes where exact string matching is commonly required. The throughput of FPGA implementation described here is essentially limited by the data transfer speed of the ATA disk drives. Higher frequency implementations utilizing Serial ATA (SATA) disk drives, parallel disk arrays, and gigabit Ethernet interfaces under investigation as part of ongoing implementation efforts and future research.

This paper also demonstrates that the significantly smaller string alphabets found in computational biology enable more space efficient designs for string matching as compared to previously published implementations focused on network intrusion detection. Although the case study focused on exact string matching, the Aho-Corasick algorithm can also accommodate regular expressions. The implementation described can easily be adapted for other types of search using, for example, spaced seeds.

The implementation presented here is restricted to searching for a maximum of 4,000 peptides. Two different approaches can be used to overcome this limitation. The simplest technique is to perform multiple passes over the string data such that a different subset of the peptides is encoded in the tiles during each pass. This approach requires only a single FPGA, but increases the overall search time by a factor given by the following expression:

(3) $$F=\left\lceil \frac{\text{total number of peptides}}{4000}\right\rceil .$$

In other words, the complexity of the search becomes *O*(*F *× (*m *+ *k*)).

A more performance oriented approach would be to use *F *separate FPGAs performing the search on separate subsets of the peptides in parallel. The cost of replicating the reading frame data for a large *F *can be eliminated by implementing a data streaming interface between the separate FPGA boards. Tools to facilitate building such interfaces are typically provided by the FPGA vendors [30,31]. Using such an interface, only one board needs to be connected to a single disk drive containing the reading frames while the other boards are connected to each other in a chain. This way, the reading frame data can be streamed from the disk drive to each board (*i.e*., as soon as an FPGA board receives a byte of data, it forwards the data to the next board in the chain). The runtime complexity of this latter implementation is essentially O(m + k + *λ*), where *λ *represents the cumulative latency of transmitting a single character over the entire chain. The *λ *value will typically be negligibly small because modern FPGAs are capable of performing serial communication at gigabit per second (or faster) rates.

## Authors' contributions

YSD developed the algorithm and implementation for string matching in FPGA hardware and conducted the experiments. SMB, SCB, and ML formulated the proteogenomic mapping pipeline problem, identified the string matching bottleneck, provided data for developing and testing the system, and assisted with the analysis of results. YSD and SMB wrote the manuscript.
